# Transcranial Auricular Vagus Nerve Stimulation (taVNS) and Ear-EEG: Potential for Closed-Loop Portable Non-invasive Brain Stimulation

**DOI:** 10.3389/fnhum.2021.699473

**Published:** 2021-06-14

**Authors:** Philipp Ruhnau, Tino Zaehle

**Affiliations:** ^1^Department of Neurology, Otto von Guericke University, Magdeburg, Germany; ^2^Center for Behavioral Brain Sciences, Otto von Guericke University, Magdeburg, Germany

**Keywords:** attention, ear-EEG, mobile EEG, non-invasive brain stimulation, taVNS

## Abstract

No matter how hard we concentrate, our attention fluctuates – a fact that greatly affects our success in completing a current task. Here, we review work from two methods that, in a closed-loop manner, have the potential to ameliorate these fluctuations. Ear-EEG can measure electric brain activity from areas in or around the ear, using small and thus portable hardware. It has been shown to capture the state of attention with high temporal resolution. Transcutaneous auricular vagus nerve stimulation (taVNS) comes with the same advantages (small and light) and critically current research suggests that it is possible to influence ongoing brain activity that has been linked to attention. Following the review of current work on ear-EEG and taVNS we suggest that a combination of the two methods in a closed-loop system could serve as a potential application to modulate attention.

## Introduction

We experience natural lapses of attention in everyday life. These fluctuations are common, and yet they can have drastic consequences if they occur in situations that require constant high attention. For instance, a major part of traffic accidents caused by human error are linked to attention lapses.

Imagine, then, if one could make use of a device that is not only capable of detecting changes in this system, but could also prevent them from occurring altogether. Such a device would have incredible potential to enhance attention in various demographics – from students learning for a test, to air-traffic control officers directing pilots safely to the airport – but also offer a variety of applications for clinical populations with attention deficits.

Current systems can detect early physiological markers of drowsiness (e.g., heart rate, respiratory activity, and eye movement) and send out warning signals to alert an individual to a lapse of attention. The fact that many vehicle companies now implement such “drowsiness detectors” or “attention assist” systems emphasizes how promising this feedback approach is. However, these approaches are still *reactive* – acting when the attention system is already fluctuating. Here, we suggest an approach that has the potential to proactively prevent such fluctuations.

This system needs to be (I) portable, to allow use in everyday life; (II) adaptable to the individual’s brain state in real time; and (III), able to stimulate the brain’s attention system non-invasively.

A system with this potential would need to comprise two parts: first, a method to read out attentive states in real time and second, a system that is capable of modulating cortical states in or close to real time. We suggest the use of the electroencephalogramm (EEG), specifically ear-EEG as a read-out and transcutaneous auricular vagus nerve stimulation (taVNS) to modulate brain activity. Combining these two into a closed-loop, would allow the three points from above to be addressed.

In the following we will provide a brief overview of work on ear-EEG and taVNS. As marker of attention, we will focus on cortical alpha oscillations. We think a closed-loop system based on ear-EEG and taVNS would provide us with a flexible, efficient, and individually tailored system that could be used to actively influence participants’ attention in real time.

## Mobile EEG and Recordings From the Ear

The first mobile EEG systems where already envisioned in the first half of the 20th century by a pioneer of EEG research, Herbert Jasper (see De Vos and Debener, 2014). Today these visions have become reality and mobile EEG systems allow for the recordings of brain activity in everyday life situations, with much greater potential to treat a variety of disorders compared to lab-based research (Debener et al., 2012). Mobile systems have been used in highly different settings, such as during physical activity (Scanlon et al., 2019), in the work space (Wascher et al., 2016), during driving (Wang et al., 2018), and even while walking a tightrope (Leroy and Cheron, 2020).

One great potential in using mobile EEG systems lies in the chance to report brain activity to the wearer and allowing for quick adjustments in behavior. This method, termed biofeedback, has used EEG to allow subjects to change their own state of mind to, for instance to alleviate stress, since the 1960s (e.g., Brown, 1974, 1977). However, this type of research, which quickly developed into treatment types, was always limited to the laboratory or clinical setting. Given that our environment is much more complex than the typical lab setting and what we can show on computer screens and present via headphones, the advantage of taking biofeedback out “for a walk” is obvious (Debener et al., 2012). The mobility of today’s EEG systems gives us the option to bring them in situations that biofeedback might be most beneficial, for instance, in anxiety inducing situations or during work where attention fluctuations can have the most impact.

A relatively novel type of mobile EEG measures electrophysiologic brain signals via electrodes connected to the ear. This can be done by either placing electrodes in the ear canal or the zymba concha (in-ear-EEG; Kidmose et al., 2012; Looney et al., 2012; Lee et al., 2014) or the area behind the ear (around-the-ear EEG/cEEGrid; Debener et al., 2015; Bleichner and Debener, 2017; Kaongoen and Jo, 2020). Major advantages of the method are its portability and unobtrusiveness – even more so than mobile EEG caps – and thereby chance to study brain activity for extended times. Ear-EEG has been suggested as a clinical tool, for instance, to diagnose epilepsy (Zibrandtsen et al., 2017; Gu et al., 2018) or to monitor sleep quality (Mikkelsen et al., 2017; Tabar et al., 2020). There are also initial results showing that levels of concentration could be monitored using ear-EEG (Kaongoen and Jo, 2020). Finally, ear-EEG has also shown to be able to track the focus of attention (Mirkovic et al., 2016) even in everyday life situations (Hölle et al., 2021).

Studies comparing ear-EEG with conventional EEG have evaluated well-known electrophysiological parameters. It has been shown that event-related potentials – such as the N1, an index of auditory sensory processing, and the P300, indexing the processing of task relevant stimuli – can be measured reliably (Looney et al., 2012; Debener et al., 2015; Mirkovic et al., 2016; Krigolson et al., 2017). Many recent developments improve the data quality that can be captured at the ear by improving the sensors or data acquisition (Kappel et al., 2019a, b; Sintotskiy and Hinrichs, 2020). Ear-EEG is most sensitive to temporal cortex activity (Meiser et al., 2020), which is great to monitor auditory system activity, such as attention to a specific sound stream (Fiedler et al., 2017). However, also dominant parietal activity, particularly neural oscillations in the alpha frequency range (around 10 Hz) can be detected well using ear-EEG (Looney et al., 2011; Debener et al., 2015; Mikkelsen et al., 2015).

Alpha brain activity is particularly interesting because it has been linked to a number of attention mechanisms and active inhibition (Jensen and Mazaheri, 2010; Klimesch, 2012; Frey et al., 2015). Most notably, attended locations are accompanied by a reduction in oscillatory alpha activity in the contralateral (compared to the ipsilateral) hemisphere that processes the location. For instance, focusing on our left hand will reduce alpha activity in the right somatosensory cortex, compared to the left, and vice versa (Haegens et al., 2011). Consequently, when alpha activity increases attention drops and subjects are more likely to miss information. Thus, an unobtrusive system that can, in real-time, measure attention drops via alpha activity reduction would open the gate to its modulation.

## Transcutaneous Auricular Vagus Nerve Stimulation

Transcutaneous auricular vagus nerve stimulation (taVNS) is a new, non-invasive neuromodulation method. TAVNS is based on electrical stimulation of cutaneously distributed vagal afferents. Unlike more established non-invasive brain stimulation methods such as transcranial direct current stimulation (tDCS) and transcranial alternating current stimulation (tACS), taVNS does not directly modulate the reactivity of neurons within specific cortical target areas. Instead, taVNS aims to promote increased noradrenergic neurotransmission through indirect stimulation of the locus coeruleus (LC), which in turn causes systemic modulation of brain function.

Transcranial auricular vagus nerve stimulation was derived from invasive vagus nerve stimulation (iVNS) that is used to treat a number of neuropsychiatric disorders (Broncel et al., 2020). IVNS is based on a neurosurgical implantation of electrodes around the left cervical vagus nerve and comes with all typical side effects associated with an invasive intervention (Fahy, 2010). The obvious benefit of taVNS over iVNS is that it is non-invasive, reducing costs and risk, and therefore having a much broader application field. It is safe and well tolerated (Redgrave et al., 2018) and has the great potential to both reduce clinical symptoms in patient populations as well as to serve basic science gain. Precisely because cost is typically a major factor that both limits access to medicine and constrains basic science, taVNS has the ultimate potential to significantly improve fairness in medical care and use in basic science.

In the last decade, there is growing evidence for a successful application of taVNS to reduce symptoms in a wide range of medical conditions including drug-resistant epilepsy and depression (Hein et al., 2013; Bauer et al., 2016), but also tinnitus (De Ridder et al., 2014), schizophrenia (Hasan et al., 2015), Alzheimer’s dementia (Kaczmarczyk et al., 2018), or chronic pain (Napadow et al., 2012). Furthermore, taVNS together with neurorehabilitation has successfully improved motor disorder symptoms in adults and children (Badran et al., 2020; Cook et al., 2020). Moreover, in healthy participants, taVNS has been proven efficient in modulating attention and cognition (Sun et al., 2017; Fischer et al., 2018; Sellaro et al., 2018). These cognitive effects of taVNS in healthy subjects and patients are assumed to be related to concentration shifts of the neurotransmitter norepinephrine (NE) and gamma-aminobutyric acid (GABA) (Van Leusden et al., 2015) caused by a stimulation of the locus coeruleus (LC) via afferent vagal fibers. For that purpose, taVNS is usually applied via electrodes attached to the cymba concha of the auricle and intends to stimulate the afferent vagal fibers of the auricular branch. TaVNS activates Aß-fibers signaling impulses ascending from the periphery to the brainstem nuclei and hereinafter to the cortex (Broncel et al., 2020; Butt et al., 2020). In particular, these vagal fibers terminate in the nucleus of the solitary tract (NTS) (Knowles and Aziz, 2009), which has widespread projections to several forebrain, limbic and brainstem sites, including the LC, the main source of noradrenaline in the human brain (Aston-Jones et al., 1991). In accordance to this anatomical connection, the core mechanism of action for taVNS relies on an activation of the locus coeruleus-norepinephrine (LC-NE) system (Frangos et al., 2015; Yakunina et al., 2017). Accordingly, in animal models and patient data, the direct link between electrical stimulation of the afferent vagal fibers and increased NE release via LC activation has been demonstrated (Dorr and Debonnel, 2006; Manta et al., 2013; Hulsey et al., 2017). Strong evidence for comparable mechanisms underlying the effects of non-invasive application of VNS in healthy participants stems from functional neuroimaging studies that consistently demonstrated taVNS induced activations in brain stem regions, including the NTS, and the LC (see Badran et al., 2018a for a recent review). Furthermore, electrophysiological marker of the LC-NE system such as the P300 component of the event-related potential (Nieuwenhuis et al., 2005; Chmielewski et al., 2017) can support the hypothesis that taVNS enhances central NE levels. Accordingly, the amplitude of the P300 is enhanced during invasive VNS (Brázdil et al., 2001; Schevernels et al., 2016) as well as during the application of taVNS (Rufener et al., 2018; Ventura-Bort et al., 2018; Lewine et al., 2019). A recent comprehensive study showed that taVNS in healthy participants systematically affected the LC-NE system indicated by a robust pupil dilation effect, accompanied by an attenuation of occipital alpha activity (Sharon et al., 2021). This study demonstrated that taVNS in healthy participants might well be able to increase attention by an elevation of noradrenaline.

Finally, animal and human studies have linked stimulation of the vagal afferent fibers to Gamma-aminobutyric acid (GABA) transmission due to activation of the NTS as well. Thus, an increase in GABA transmission can be assumed as a secondary mechanism of action of taVNS (Keute et al., 2018; Broncel et al., 2019). Alternatively, it has been assumed that taVNS always has a combined effect on both, NE and GABA (Beste et al., 2016).

Although attention is regulated by several neurotransmitter systems, noradrenaline is one of the most important. Accordingly, central noradrenaline is involved in the control of attention (Woodward et al., 1979), and further plays an important modulatory role in cognitive processes such as vigilance, arousal, learning, and memory (Aston-Jones et al., 1991). It has been consistently shown that reducing central NE had deleterious effects on attention (Smith and Nutt, 1996), while elevating central NE improved performance in attention tasks (Sirviö et al., 1993; Bunsey and Strupp, 1995), indicating that an increased NE activity facilitates cortical circuit function that promote alertness and attention (Aston-Jones and Cohen, 2005).

Animal and human data further show that NE maintains an active role in regulating sustained and flexible attention (Aston-Jones et al., 1997; Aston-Jones and Cohen, 2005). Analogously, in healthy humans noradrenergic manipulation impairs sustained attention (Coull et al., 1995; Smith and Nutt, 1996), as well as focused and divided attention during dichotic listening experiments (Clark et al., 1986).

GABA is the main inhibitory neurotransmitter in the adult mammalian brain, but its role in the regulation of arousal and attention is less clearly defined. However, several data point to the notion that GABA plays an important role in the regulation of attention as well (Paine et al., 2015; Leonte et al., 2018). Animal data demonstrate a direct link between GABA levels in the brain and visual attention (Petersen et al., 1987) and relate the activity of GABAergic neurons to the regulation of attention (Paine et al., 2011; McGarrity et al., 2017). Consequently, decreasing GABA functioning impairs visual attention (Paine et al., 2011) while sub-optimally increasing it impairs attentional processing as well (Pezze et al., 2014).

Thus, attentional functions are strongly dependent on noradrenergic and GABAergic transmission. TaVNS has been demonstrated to be efficient in modulating these neurotransmitters and, accordingly directly affecting attentional processes.

## Combining taVNS and In-Ear EEG

**FIGURE 1:**
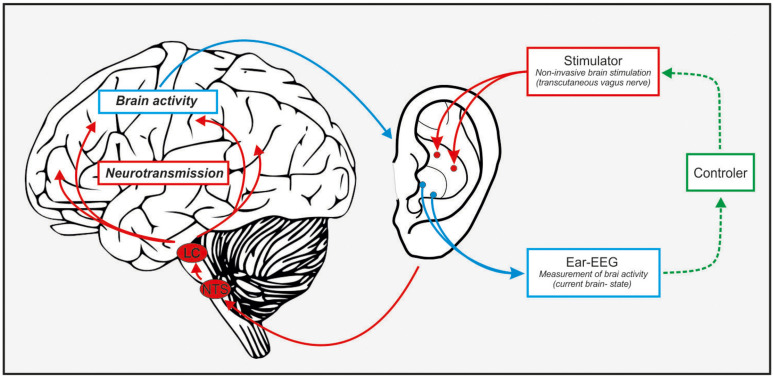
Schematic illustration of a closed-loop in-ear stimulator/recorder. Ear-EEG can pick up on attention markers (alpha oscillations, event-related P300), this information gets fed to a controller that – given a decrease in attention – can start the taVNS which stimulates the NTS-LC system and elevate NE levels. This in turn should result in an increase of attention (or a reduction of the decrease).

We suggest that combining the two mentioned methods offers a great opportunity for a portable, closed-loop, monitoring, and non-invasive stimulation device to stabilize fluctuations of sustained attention in a brain-state dependent manner (see Figure 1 for a schematic illustration). Here, in-ear EEG will provide the biomarker-feedback signals that, in turn, modify stimulation parameters based on the adaptive feedback signal (closed-loop).

The EEG signal provides reliable markers of attention states; such as fluctuations in the cortical alpha rhythm that mirror attentional fluctuations (Fiedler et al., 2017; Jeong and Jeong, 2020). The ear-EEG system gives subjects the opportunity to move freely and wear the device for long periods of time. At the same time brain activity can be recorded at high temporal precision with the opportunity to transmit these data wirelessly (Bleichner and Debener, 2017; Kaveh et al., 2020). This would allow for constant monitoring of the participant’s attention state. The ear-EEG readout can be easily processed by small controllers which could in turn provide information to the wearer.

Electroencephalogramm brain signals have been used directly as informative biofeedback in closed-loop systems, for instance, to reduce an imbalance of brain rhythms in tinnitus (Hartmann et al., 2014), as a treatment for epilepsy (Tan et al., 2009), or to improve symptoms in attention deficit disorder (Monastra et al., 2002). Sending feedback to the participant based on brain activity is powerful and fast, yet it is only slightly faster than measuring and reporting peripheral physiology (e.g., drowsiness detectors in cars, García et al., 2010; Leonhardt et al., 2018). The substantial improvement needed is a fast track to brain activity underlying attention lapses. This fast track could be provided by non-invasive transcranial brain stimulation, such as taVNS, which as reviewed above can successfully modulate brain activity via the LC-NE route. This non-invasive stimulation technique directly influences the attention network in the brain (Fang et al., 2016) and is therefore an ideal partner of ear-EEG. Furthermore, it has been shown that alpha oscillations can be modulated via taVNS (Sharon et al., 2021). Therefore, a closed-loop ear system focusing on alpha activity seems very promising.

A major advantage of the closed-loop system is that it respects state-dependent efficacy of non-invasive brain stimulation (Bergmann, 2018). It has been shown that transcranial electric stimulation of a specific neuronal population is more effective when applied when the region is active. For instance, stimulation is much more successful when targeting a region engaged in a task (Alagapan et al., 2016; Li et al., 2018) while stimulation efficacy of highly active regions is limited (Ruhnau et al., 2016). So far this has not been evaluated for taVNS and further the mechanism of action is rather global, instead of region specific for tACS or tDCS, therefore future research is essential to uncover state-dependent efficacy of taVNS.

There are a number of attention trainings that improve attention function and generalize to other cognitive functions (for a review see Tang and Posner, 2009; for a meta-analysis see Peng and Miller, 2016). It is important to compare effects of a closed-loop system to those of training programs to evaluate the benefit of such a system. Furthermore, a combination of taVNS and ear-EEG with attention trainings could be a fruitful avenue that might improve the benefit from the training. As mentioned before, brain stimulation depends on the brain state, thus applying stimulation during a training of attention might be even more beneficial.

In our view, alpha activity fluctuations are the best candidate at the moment to read out attentional states, they can be extracted in real time, and can be directly modulated non-invasively via taVNS. Consequently, groups that are affected by reduced alpha activity are ideal targets for the proposed closed-loop system. There are a number of disorders that can be linked to dysfunctional alpha activity compared to healthy control groups (for a review see Başar, 2013), such as depression (Jiang et al., 2016; Alexander et al., 2019), attention deficit disorder (Hasler et al., 2016), Alzheimer’s (Jafari et al., 2020), and Parkinson’s disease (Zhu et al., 2019).

Furthermore, closed-loop EEG-VNS system can be of use in healthy populations in situations where a high and constant level of attention is required, for instance during aviating or driving. The great potential of this closed-loop system is the ongoing and extremely fast measurement of the brain state and immediate intervention when an attention lapse is approaching.

It is important to mention that research on brain stimulation has shown long-term plasticity effects due to stimulation. That means a permanent application of the closed-loop system is neither necessary nor intended, because stimulation-based functional and anatomical changes will improve attention abilities and reduce symptoms in patients following short-term application.

## A Need for Parametrization of taVNS Settings

We want to take this opportunity to point out that taVNS results have recently shown variable efficacy of the method. There have been a number of studies providing inconsistent results of efficacy of taVNS, either absent effects or even inverted effects (e.g., Keute et al., 2018, 2019; Borges et al., 2021). One reason for this might be the variability of taVNS stimulation parameters in use. There have been a number of different stimulation frequencies (between 0.5 and 30 Hz), pulse width (50–500 μs), intensities (0.5–50 mA) and stimulation locations (see Burger et al., 2020 for a list of parameters). Very few studies aim to evaluate the role of taVNS parameters on efficacy. For instance, recently a study (Sharon et al., 2021) showed that taVNS when applied for a short time (3 s) can influence the pupil size – an index of LC driven NE level modulation – which has not been found for a more typical long-interval (30 s on/off) taVNS protocol (Keute et al., 2019). Unfortunately, there are no studies as of yet that evaluate which parameters influence taVNS efficacy systematically. Thus, at the moment we have no knowledge whether it is the duration of taVNS as suggested by Sharon et al. (2021) or any other of the parameters such as intensity, pulse width, or electrode size, which were all different in the two studies.

A promising approach might be to relate other physiological parameters that have been connected to vagal stimulation to neurostimulation efficacy. As such, the taVNS efficacy in modulating the heart rate (Badran et al., 2018b) and coupling of cardiac to neural activity seem promising (Keute et al., 2021) but warrant further investigation as well.

As with any new approaches in neuromodulation there is an urgent need to evaluate how to set the stimulations to yield maximal effects. This is not just an issue for taVNS but for the whole field of transcranial brain stimulation (Parkin et al., 2015; Frohlich and Townsend, 2021). One way to evaluate the effects of different parameters is to study efficacy of taVNS in a closed-loop as we proposed here. It would allow us to monitor effects in real time and adjust the stimulator accordingly, homing in to maximal efficacy.

## Limitations and Challenges

An obvious challenge for a combination of taVNS and ear-EEG are their close proximity in the (most likely same) ear. For the EEG this means that there is unlikely a recordable signal, while the stimulation is running. Thus, the impact on the stimulation on brain activity can only be measured with a delay. Designs similar to Sharon et al. (2021) that use short taVNS trains followed by no-stimulation intervals will help investigate the stimulation effects in the closed-loop system.

Another challenge is posed by the size of the ear, which limits space for electrodes. It remains to be tested whether similar locations can be used for stimulation and recording (in the ear canal, for instance) or which placements of recording and stimulating electrodes is the most feasible, following space constraints, and the most effective. A combination of taVNS and cEEGrids (Bleichner and Debener, 2017; Meiser et al., 2020) seems promising because sensors and stimulator electrodes would be apart by design.

It is, furthermore, critical that the link between the measured alpha activity with ear-EEG and a potential attention drop is further investigated. Previous research showed that alpha power preceding weak visual stimuli can predict detection performance (e.g., Hanslmayr et al., 2007; van Dijk et al., 2008; for a review see Ruhnau et al., 2014). The causal involvement of alpha power in visual attentive states has been further confirmed using transcranial magnetic stimulation (Romei et al., 2011). However, how well ear-EEG can pick up these alpha fluctuations linked to (dominantly visual) attention, remains an open question. Thus, before a closed-loop system can leave the lab, an evaluation *in the lab* is essential. For instance, future studies should evaluate if ear-EEG signals in the alpha range can predict visual perception in near-threshold cases (such as in Hanslmayr et al., 2007; van Dijk et al., 2008). Given that alpha power lateralization is often a good predictor (Thut et al., 2006), ear-EEGs on the left and right ear might be essential to test this lateralization properly.

Moreover, it is important to emphasize that keeping attention at a high level for an extended time is not beneficial, nor intended with the system we propose. There are time limits how long people in different professions are allowed to work before they are required to take a break. For instance, in the EU professional drivers are not allowed to drive a vehicle longer than 9 h per day (with few exceptions), or interpreters working for the United Nations are required to take at least one and a half hour breaks between maximally 2.5–3 h work sessions. Maintaining high levels of focus is tiring and requires rest. Hence, an application that we suggest, should not aim to increase the duration on a task but rather normalize attention fluctuations while on the task.

Typical mobile stimulators contain constraints as to the specific settings of the stimulation (tailored to the individual user) and the daily dose. These factors also need to be investigated and kept in mind when using taVNS and ear-EEG together. Given that taVNS is still a relatively novel non-invasive brain stimulation tool, it is important to properly record and monitor short term as well as long term side effects and adjust protocols to avoid them as well as possible. Similar standards as suggested for other transcranial stimulation techniques might be chosen as a baseline (for comprehensive guidelines see Rossi et al., 2009; Antal et al., 2017) but data on unwanted effects needs to be carefully collected and monitored.

## Conclusion

In summary, the use of a closed-loop system consisting of ear-EEG and taVNS holds the promise of a potential therapeutic option in neuropsychiatric patients as well as a supportive device in healthy populations. Therefore, we highly encourage to explore the usability of such a closed-loop system.

Further research is needed to determine the exact parameters of optimal taVNS to fully exploit its potential. Research efforts will need to focus on the systematic investigation of suitable parameter settings, especially stimulation duration, to maximize efficacy as well as long-term effectiveness.

## Author Contributions

PR and TZ wrote the manuscript and prepared the artwork. Both authors contributed to the article and approved the submitted version.

## Conflict of Interest

The authors declare that the research was conducted in the absence of any commercial or financial relationships that could be construed as a potential conflict of interest.
